# Indigenous food production in a carbon economy

**DOI:** 10.1073/pnas.2317686121

**Published:** 2024-07-29

**Authors:** Elspeth Ready, Cody T. Ross, Bret Beheim, Jenn Parrott

**Affiliations:** ^a^Department of Human Behavior, Ecology and Culture, Max Planck Institute for Evolutionary Anthropology, Leipzig 04103, Germany; ^b^Department of Anthropology, University of Florida, Gainesville, FL 32611; ^c^Innovation, Inuvialuit Science & Climate Change Division, Inuvialuit Regional Corporation, Inuvik, NT X0E 0T0, Canada

**Keywords:** Inuit, climate change policy, carbon emissions, Indigenous food systems, sustainability

## Abstract

Indigenous communities in the North American Arctic are characterized by mixed economies that feature hunting, fishing, gathering, and trapping activities—and associated sharing practices—alongside the formal wage economy. The region is also undergoing rapid social, economic, and climate changes, including, in Canada, carbon taxation, which is impacting the cost of fuel used in local food harvesting. Because of the importance of local foods to nutrition, health, and well-being in Arctic Indigenous communities, there is an urgent need to better understand the sensitivity of Arctic food systems to social, economic, and climate changes and to develop plans for mitigating potential adverse effects. Here, we develop a Bayesian model to calculate the substitution value and carbon emissions of market replacements for local food harvests in the Inuvialuit Settlement Region, Canada. Our estimates suggest that under plausible scenarios, replacing locally harvested foods with imported market substitutes would cost over 3.1 million Canadian dollars per year and emit over 1,000 tons of CO_2_-equivalent emissions per year, regardless of the shipping scenario. In contrast, we estimate that gasoline inputs to harvesting cost approximately $295,000 and result in 315 to 497 tons of emissions. These results indicate that climate change policies that fail to account for local food production may undermine emissions targets and adversely impact food security and health in Arctic Indigenous communities, who already experience a high cost of living and high rates of food insecurity.

## Significance

Local foods are critical to the food security and health of Indigenous peoples around the world, but the importance—both monetary and environmental—of local “informal” economies is often not visible to policymakers. Here, we combine data from multiple sources and use Bayesian inference techniques to estimate the carbon emissions that would be produced by market replacements for local food in the Inuvialuit Settlement Region, Canada. We show that Inuit harvesting is more carbon efficient than importing market substitutes, in addition to being less reliant on vulnerable supply chains. These findings highlight the importance of place-based and culturally-informed approaches to climate policy for remote and Indigenous communities.

Local food harvesting, and associated cultural practices, including food sharing and craft production, generates significant economic and social value in many Indigenous communities around the world. In the Arctic, these practices, often referred to as the “subsistence” or “traditional” component of mixed economies, support food security and nutrition, build trust and social capital, and promote both physical and mental health. Because of their nonmonetary nature, these traditional economies are largely invisible to national- and territorial-level economic statistics, but they are both economically important and culturally salient in communities throughout the North American Arctic.

Economic, social, or environmental changes that impact access to wildlife resources are likely to have negative nutritional, health, and cultural impacts on remote Indigenous communities. Although resource flows in subsistence economies are less easily measured than flows of cash, attempts to quantify their outputs have a long history in both northern Canada and Alaska (1–3). Resource considerations alone cannot capture the full meaning and value of traditional harvesting activities for northern Indigenous peoples. Nevertheless, estimates of local food harvests and their monetary value have been an important source of information assisting both governments and local rightsholders in negotiating wildlife management policies, conducting environmental impact assessments, and developing food security and nutrition policies throughout Northern Canada and Alaska for decades.

Past assessments of Arctic harvest production have focused on estimating the edible weight of locally harvested food and its substitution value (i.e., the cost of purchasing an equivalent amount of market food). However, additional metrics are relevant today. Carbon pricing has been in effect throughout Canada since 2019, impacting the price of many goods and services, particularly fossil fuels. Further increases in carbon prices are scheduled to occur over the next several years (4). While the Canadian federal government has committed to avoid placing a disproportionate burden on Indigenous peoples as a result of carbon pricing policies (5), an understanding of how the traditional economies of northern regions will be affected by such policies, and a clear plan for mitigating any potential impacts, are still lacking.

What are the likely impacts of a transition to a low-carbon economy for Arctic food systems? Indigenous harvesters in northern Canada and Alaska rely on fossil fuel-powered transport (e.g., boats, snowmobiles, and all-terrain vehicles). Alternative modes of transport, including electric vehicles, are not currently viable given the remote location and climate. Rates of food insecurity are also high in Arctic communities, a problem related to the high cost of imported foods. Under these conditions, what sorts of policies could promote decarbonization without undermining the persistence and sustainability of local harvesting practices and food security? As a first step toward addressing these questions, we focus on calculating the edible weight of harvested foods and determining its substitution value in the Inuvialuit Settlement Region (ISR). We extend past work on this topic by estimating not only the substitution value of wild harvests, but also the carbon emissions associated with the importation of substitute market foods to Arctic communities. We also generate some initial estimates of the carbon inputs to subsistence harvesting, focusing on gasoline usage. We accomplish this by developing a bespoke Bayesian analysis of data on harvesting and trapping activities for the six communities in the ISR, and pair the results of that analysis with estimates of carbon emissions for the production and transport of comparable market foods gleaned from agricultural and transportation science research.

## Background: The Inuvialuit Traditional Economy

**Fig. 1.**
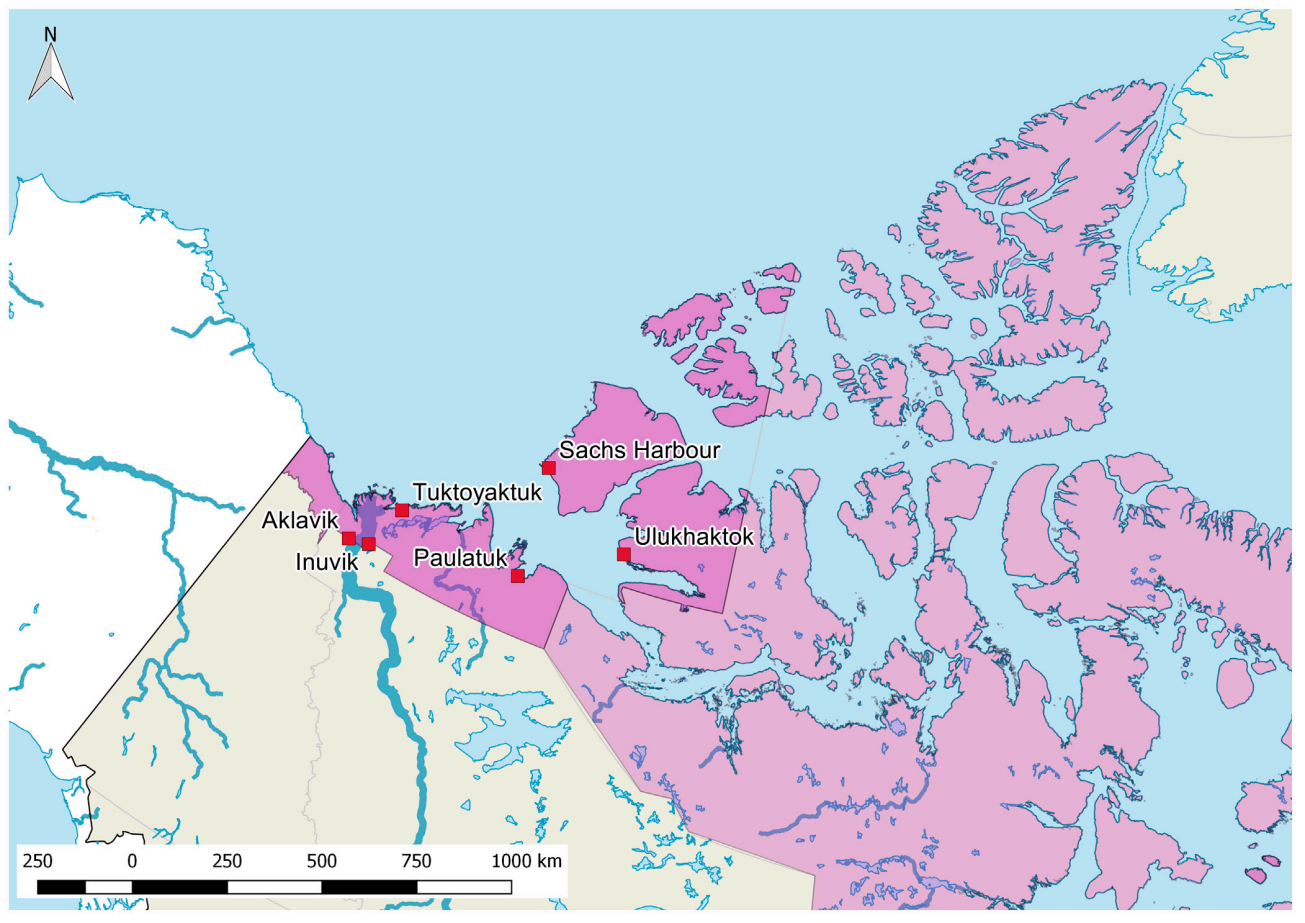
Locations of the six study communities are plotted in red. The ISR is highlighted in dark pink. The light pink region shows other regions of Inuit Nunangat in Canada.

Many of the challenges faced by residents of northern settlements in North America are similar to those faced by residents of remote rural regions around the globe, including unreliable supply chains, colonial histories, distributed energy infrastructure, and limited economic development (6). Our focus here is on the ISR, which is the westernmost Inuit region in Canada (Fig. 1). The region comprises six settlements with approximately 5,300 residents, roughly 2,800 of whom are Beneficiaries of the Inuvialuit Final Agreement. Inuvik (the largest settlement) and Tuktoyaktuk are accessible by road for most of the year, via the Dempster and Inuvik-Tuktoyaktuk highways.

As a result of their remote location, ISR communities have a high cost of living and a heavy dependence on fossil fuels for travel, transport of goods, heating, and electricity (7, 8). Modern harvesting, fishing, and trapping methods also depend on a wide range of imported equipment and supplies, including rifles, boats, snowmobiles, and gasoline. The interdependence of the cash and subsistence economy in the North American Arctic has developed over the course of more than a century and has enabled subsistence harvesting to persist as a vital component of Inuit livelihoods, despite decades of centralized settlement (9). However, in recent decades, the cost of equipment and supplies has become a major barrier to local food production for Inuit in Canada (10, 11). Amid these ongoing challenges, climate change is also creating new conditions to which Arctic harvesters must adapt.

Inuvialuit today engage in a wide range of harvesting activities on land, water, and ice. More than 50 animal species are harvested in the ISR, including birds, fish, and both land and sea mammals. The vast majority of animals harvested are used for food; those that are not used as food are predominantly small carnivores (e.g., fox, mink) whose furs are sold. As elsewhere in Inuit Nunangat, rates of food insecurity in the ISR are extremely high. The 2017 Aboriginal Peoples’ Survey found that 49.9% of Inuvialuit were moderately to severely food insecure, a rate nearly 5.7 times higher than in Canada as a whole (12). An important contributor to the high rate of food insecurity in the ISR is the high cost of imported foods, which persists despite federal subsidy programs (13, 14). Locally-harvested traditional foods constitute roughly 16% of the total calories consumed by Inuvialuit, and represent a major source of protein, iron, niacin, and vitamins D, B6, and B12 (14). The nutritional importance of traditional food is related to the high cost of nutrient-rich store foods, along with high rates of poverty in Inuit communities (14–17). High intake of non-nutrient-dense foods with high fat or sugar content is linked to food insecurity, obesity, cardiovascular disease, and diabetes among Inuit (18–24).

The redistribution of traditional foods between households plays a key role in buffering individuals against food insecurity and malnutrition. Traditional food sharing practices can improve overall community-level food security through flows of resources from high-producing households to low-producing households (25, 26). The redistribution of food also provides value through the social ties it creates and reinforces: by sharing food, information, and equipment, households build relationships of trust that can help them deal with future challenges (27). The value of this social insurance is extremely difficult to measure, however, because it is embedded in social relations and in their capacity to reorganize over both short-term and long-term time scales.

Finally, the benefits of Inuit traditional economies reach beyond the domains of nutrition and food security. At the community level, Inuit traditional economies help build trust, facilitating community projects and collective decision-making (28–30). Adherence to the cultural values and behaviors that permeate the traditional economy promotes resilience to stress (31, 32) and helps to mitigate the risk of substance dependence and adverse mental health outcomes (33–35). The traditional economy thus supports Inuit mental and physical health without requiring substantial inputs of government money and infrastructure. This contribution is particularly valuable given the geographic, cultural, and infrastructural barriers that Inuit face in accessing health services (36).

As part of an effort to protect and promote the well-being of Inuvialuit residents, it is therefore important to examine the potential impacts of climate change and climate change policy on the Inuvialuit Traditional Economy. Of particular importance is the ongoing implementation of carbon pricing and net zero carbon policies in Canada. Here, we aim to provide insight into the sustainability of current Arctic mixed economies by assembling data from a wide variety of sources and developing statistical models that allow us to estimate the “carbon value” of Inuit food production. While carbon storage through Indigenous protection of forest reserves has previously been demonstrated (37), this study attempts to quantify carbon emissions avoided or incurred through Indigenous food production.

## Results

### Total Harvest Estimates.

**Fig. 2.**
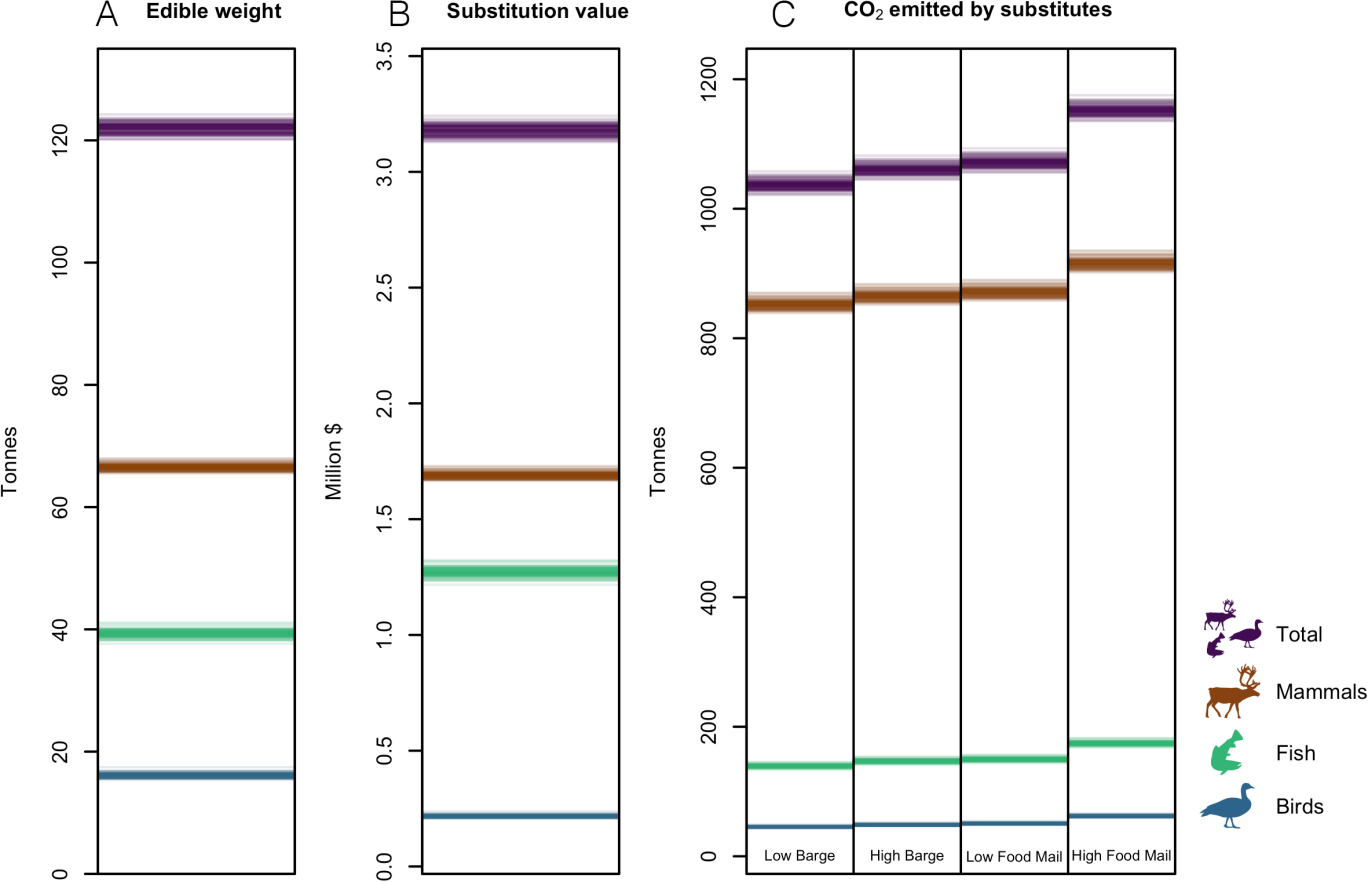
Posterior estimates of (*A*) the total edible weight of the harvest reported in the 2018 IHS, (*B*) the substitution value of the reported harvest, and (*C*) the greenhouse gas emissions (CO_2_-equivalents) of market substitutes, showing estimates for four emissions scenarios (barge shipping at low and high emissions projections, and food-mail shipping, also at low and high emissions projections). Each semitransparent line (*n* = 100 for each estimand) represents one draw from the posterior distribution such that darker shading indicates higher density of estimates.

Fig. 2*A* and Table 1 present the estimated edible weight of harvested food represented by the 2018 Inuvialuit Harvest Study (IHS) data. The reported 2018 harvest totals 122,117 ± 886 (mean, SD) kilograms of food. The most important food species by edible weight are caribou (21,430 ± 297 kg), broad whitefish (12,737 ± 316 kg), muskox (11,460 ± 233 kg), and inconnu (10,627 ± 470 kg). The total reported harvest corresponds to an average of 44.1 kg per resident Inuvialuit Beneficiary. Harvest estimates range from a high of 95.9 kg per Beneficiary in Ulukhaktok, to a low of 16.2 kg per Beneficiary in Inuvik. However, our estimates represent only a portion of the “true” total harvest, as survey participation rates suggest that reported harvests represent as little as 50% of the total harvest in some communities (*SI Appendix*).

**Table 1.** Estimated edible harvest reported in the 2018 IHS

	Mean (kg)	SD	90% HPDI (low)	90% HPDI (high)
Birds	16,211	372	15,641	16,747
Fish	39,308	608	38,410	40,222
Mammals	66,597	549	65,668	67,477
Total	122,117	886	120,573	123,407

90% HPDI is the highest posterior density interval, the smallest interval within the posterior distribution that accounts for 90% of posterior samples.

### Market Replacement Cost.

Fig. 2*B* and Table 2 summarize the estimated replacement value of food reported in the 2018 IHS. Our estimate for the substitution value of the reported harvest is approximately 3.18 ± 0.02 million Canadian dollars. This corresponds to roughly $1,150 per Inuvialuit Beneficiary, ranging from $315 per Beneficiary in Inuvik to $2,802 per Beneficiary in Ulukhaktok. Again, these estimates are based only on the amount reported in the 2018 IHS and are thus a lower bound for the total harvest in the region. If we additionally account for food subsidies in the more remote communities, the amount of food reported in the IHS would result in an additional cost of approximately $394,546 (*SI Appendix*).

**Table 2.** Estimated substitution value of the reported harvest, in 2018 Canadian dollars

	Mean ($)	SD	90% HDPI (low)	90% HDPI (high)
Birds	218,506	5,295	210,343	226,056
Fish	1,270,756	18,916	1,241,336	1,299,060
Mammals	1,691,827	14,194	1,668,580	1,715,630
Total	3,181,089	23,970	3,141,689	3,218,131

### Carbon Emissions of Market Replacements.

Fig. 2*C* and Table 3 report the carbon emissions that would be incurred through the production and importation of market substitutes for the harvests reported in the 2018 IHS. We report four estimates: “low” and “high” emissions projections based on the range of published emissions estimates for each transportation mode, for each of two different transport scenarios. The “barge” scenario represents a case in which foodstuffs are brought by road to Hay River and then by barge to Inuvialuit communities, while the “food mail” scenario represents a case in which foodstuffs are imported by road to Inuvik and Tuktoyaktuk and then flown into the smaller communities. Our analysis suggests that a quantity of food equivalent to the harvests reported in the 2018 IHS would produce over 1,000 tons of greenhouse gases per year, regardless of the mode of transport. The mean carbon emissions estimates for each transport scenario suggest that, per kilogram of country food consumed, 8.5 to 9.4 kg in gross CO_2_-equivalent emissions are avoided, or between 375 to 417 kg CO_2_-equivalent emissions per year per Inuvialuit Beneficiary (we consider carbon inputs to the traditional economy in the subsequent section). Once again, this is an estimate based on the reported, and not the true total, harvest.

**Table 3.** Estimated carbon emissions (CO_2_-equivalents) of market substitutes for harvests reported in the 2018 IHS, in metric tons

	Birds			Fish			Mammals			Total		
	5%	Mean	95%	5%	Mean	95%	5%	Mean	95%	5%	Mean	95%
Low barge	44	46	47	136	139	143	841	853	864	1,026	1,038	1,050
High barge	47	49	50	144	147	150	854	866	877	1,050	1,062	1,074
Low food mail	49	51	53	146	150	153	860	872	883	1,060	1,073	1,085
High food mail	60	62	64	171	174	178	904	916	929	1,140	1,153	1,166

5% and 95% columns indicate the lower and upper bounds of the 90% HPDI (highest posterior density interval). We compare four scenarios: low- and high-emissions projections for food shipped by road/barge and for food shipped by food mail (*SI Appendix*).

### Gasoline Inputs to Local Production.

Using parameters estimated in a regression analysis of the Tooniktoyok dataset, we estimate that 126,953 ± 15,303 L of gasoline would have been used in producing the harvest reported in the 2018 IHS. We further estimate an associated 781 ± 173 unsuccessful trips associated with the reported harvest, which would have consumed an additional 40,408 ± 15,668 L of gasoline. Thus, the total fuel consumption estimates for the harvest reported in the 2018 IHS is 167,362 ± 21,957 L. This corresponds to roughly 1.4 L of gasoline per edible kilogram harvested. Using $1.76 a liter as an approximation for the cost of fuel in 2018 (38), this translates to a total of $294,557 ± 38,644 in gasoline costs for the reported harvest, or roughly $2.41 per kilogram harvested. The Tooniktoyok data suggest that gasoline represents roughly 50% of the cost of supplies for harvesting trips (*SI Appendix*). Therefore, excluding major equipment costs, per trip expenses for harvesting are well below the replacement cost of market foods (which average $26.04/kg). Carbon emissions associated with the gasoline used in harvest production reported in the IHS range from 315 to 497 tons (low-emissions scenario 90% HPDI: 315 to 479 tons; high-emissions scenario 90% HPDI: 327 to 497 tons). Mean posterior estimates from these scenarios correspond to 3.3 kg (low) or 3.4 kg (high) of carbon emissions per kilogram of food produced and 145 kg (low) or 150 kg (high) of CO_2_-equivalent emissions per Inuvialuit Beneficiary.

## Discussion

The Inuvialuit Traditional Economy is an economic system based on the harvesting, sharing, and use of wildlife that has persisted despite the disruptive impact of settlement and integration into the cash economy over the last century. However, Inuvialuit harvesting activities today are heavily dependent on gasoline, a dependence that is rooted in colonial policies. Given the remote location and climate of the ISR, and the needs of the current population, this dependence on gasoline cannot be easily reduced with currently available technology. Arctic communities, like remote communities elsewhere in the world, are much more constrained in their ability to decrease fossil fuel reliance than urban, better connected, or more densely populated regions. The Arctic is also at a major competitive disadvantage in the global economy, an issue which is reflected in the high cost of living, limited economic opportunities, and high rates of poverty in Inuit Nunangat.

Our analysis indicates that approximately 122 tons of food, with a retail substitution value of over 3.18 million dollars, are represented in the 2018 IHS data. Comparison of our results with past estimates of the harvest in the Inuvialuit region suggests that harvest rates have remained stable in recent decades (*SI Appendix*). If commercially farmed meats shipped by food mail were substituted for this quantity of food, they would produce over 1,000 tons of CO_2_-equivalent emissions, or 375 to 417 kg CO_2_-equivalent per Inuvialuit Beneficiary, depending on the transport scenario. For comparison, the average yearly carbon footprint for people in Canada is 15.50 tons, and the average worldwide per capita carbon footprint is about 4.79 tons (data from https://www.carbonfootprint.com/calculator.aspx).

Gasoline consumed in the production of local harvests results in carbon emissions that are approximately one-third to one-half as large as those of an equivalent amount of imported market foods. Due to a lack of data, we could not calculate a precise estimate for the carbon emissions related to the production and shipping of vehicles used in harvesting, which are the other main source of emissions incurred through harvesting. Nevertheless, it is clear that the Inuvialuit Traditional Economy dramatically reduces the need for food to be inefficiently transported from southern markets, especially when considering that the true amount of food harvested in the ISR in 2018 is likely to be considerably higher than the conservative estimates reported here.

Our findings illustrate how local food harvesting, even when reliant on fossil fuels—as is the case in Canadian Arctic communities—can be more economically efficient and less carbon intensive than industrial food production. Local food harvesting also reduces reliance on supply chains that are vulnerable to climate change. As a case in point, in the summer of 2023, goods en route to the Inuvialuit Region had to be shipped an extra 4,000 kilometers due to low water levels on the Mackenzie River (39). Ensuring the food security of Indigenous peoples and achieving decarbonization objectives therefore requires acknowledging the economic and cultural importance of Indigenous food systems.

Unfortunately, while current carbon pricing policy includes subsidies for food imported to Inuvialuit communities and exemptions for farming and fishing industries, it does not include any exemption or support for Indigenous subsistence harvesters. Our findings highlight the economic and cultural risks of such policies, emphasizing the need for climate action that is place-based and that accommodates the knowledge and values of local rightsholders. In Inuit Nunangat, carbon tax measures will lead to increases in the costs of harvesting supplies (such as gas) and equipment, which is already a significant obstacle for many Inuit (10, 11). This may have the perverse effect of increasing carbon dependence, not only by increasing reliance on imported substitutes, but also by reducing subsistence participation in Indigenous communities. Carbon tax measures not adapted to the particular needs of remote Indigenous communities will lead to other kinds of costs, including the social costs that might result from weakened cooperative hunting, food-sharing, and information exchange networks, and the increased need for healthcare services that might result from increased dependence on imported calorie-dense, but nutrient-poor, food items.

By increasing the cost of traditional harvesting, carbon pricing potentially threatens Inuit harvest production and the numerous kinds of value—both economic and cultural—that it produces. High carbon tax rates may prevent hunters from responding adaptively to changes in their environment. For instance, harvesters may need to travel more in certain years to collect information about a changed environment, but such trips may become economically unfeasible. Protecting the ability of harvesters to react flexibility and dynamically to their local environment is an important component of maintaining harvest production and reducing dependence on imported foods. Similarly, if harvesting decreases, the weakening of social networks produced and maintained through cooperative harvesting and food-sharing may also disrupt community social capital, and reduce the capacity of communities to respond to other new and ongoing stressors—including those related to climate change. Our results provide a clear example of why effective and equitable climate policy for remote regions needs to be informed by local cultural, economic, social, and environmental factors. Developing such policies requires engagement with local knowledge and rights holders.

## Materials and Methods

### Research Design and Permissions Process.

This project was initiated by the Innovation, Inuvialuit Science, and Climate Change Division of the Inuvialuit Regional Corporation (IRC), whose mission is to coordinate research and develop policies for the ISR. The IRC is an Inuvialuit-led organization mandated to improve the economic, social, and cultural well-being of Inuvialuit through the implementation of the Inuvialuit Final Agreement, which was one of the first modern land claims agreement in Canada. E.R. was initially contracted by the IRC to produce a report estimating the size of the Inuvialuit Traditional Economy (40), as part of a series of reports focused on assessing the potential impacts of carbon tax policy on Inuvialuit Beneficiaries. After completion of the report, E.R. proposed publishing the results in a scientific journal, to disseminate the findings more widely. This process involved submitting a formal proposal to the IRC, who evaluated the potential costs and benefits of such a publication for Inuvialuit. The proposal and the subsequent manuscript were reviewed and approved by the IRC.

The data used in this study originate from two main sources, access to which were granted by the IRC and other relevant parties (*Data, Materials, and Software Availability*). The first data source, the IHS, is a long-term data collection initiative led by the IRC in collaboration with the Hunters and Trappers Committees (HTCs) in the six ISR communities. The second data source is the Tooniktoyok Project, a community-based research project conducted in Ulukhaktok in 2019 (41, 42).

### Harvest Data.

We use 2,388 harvest reports from the 2018 IHS to estimate the quantity of food harvested in the ISR. The IHS is administered locally in each community in the Inuvialuit region by HTCs. Only members of the HTCs were included in the study, and participation in the study was voluntary. In principle, local interviewers contacted all HTC members each month to record all harvests and determine whether any harvesters were inactive or “out-of-sample” (e.g., away from town) for a given month. For a variety of reasons, the participation of HTC members in the 2018 survey was relatively low (close to 50% on average) and variable from month to month. For example, in some months, in some communities, no harvests were reported at all due to a lack of available interviewers. Due to these data-quality issues, extrapolating from the reported harvest to an estimate of the total harvest is not straightforward. In view of this, we emphasize that we focus on calculating the harvest reported in the IHS, which we take as a minimum estimate for the total harvest that year. In supplementary materials, we examine the data on participation rates in the 2018 IHS.

The harvest data in the IHS are based on participant recall. Because Inuit harvesters rarely count their catch, their estimates are approximate and strongly heaped (e.g., catches of several animals tended to be reported in round multiples of 5, 10, or 50). Below we detail the statistical procedure we use to account for this feature of the data. Also, due to similarities in variable names in the data collection platform, harvester IDs were sometimes recorded under the harvest number variable. To correct for these data-entry errors, we filtered the data to identify likely cases (*n* = 64) and flagged the harvest amount as missing. We then impute the missing harvest amounts using Bayesian methods during the modeling procedure.

Our modeling approach was also influenced by the fact that the IHS interviews focus only on harvests, and, as such, there is a lack of information about unsuccessful harvesting trips. Although unsuccessful trips are not required for estimating the total harvest, they are needed to estimate the cash inputs to and carbon outputs from harvesting. To deal with this issue, we use the Tooniktoyok dataset (described below) to generate estimates of the number of unsuccessful harvest trips and gas consumption associated with them, and then we adjust the cash and carbon estimates for the observed harvests using these rates.

### Edible Weight Data.

Once harvest quantities are estimated, they need to be converted to edible weights. To obtain edible weight data, we reviewed three main sources: Usher’s calculation of edible weights for species harvested in the ISR (43); Ashley’s review of all available edible weight estimates for game in the NWT and Nunavut (44); and Brown et al.’s derivations of new estimates for several fish species caught in Alaska (45). Our list of edible weight values, including justifications for which values we adopted when multiple values were available, as well as descriptions of calculations for values not available in the published literature, are provided in *SI Appendix*.

### Substitution Value.

Price estimates of the cost per kilogram of replacement foods (*SI Appendix*, Table S1) are based on average prices reported in each ISR community from an in-store pricing study (14), which established the lowest regular prices for preferred purchase volumes of a wide variety of foods. We group these products into three broad categories: mammals, poultry, and fish. For mammals, we use a mix of 50% pork and 50% beef (see *SI Appendix* for details and justification). For poultry, we use the price of an even mix of chicken legs and chicken breasts. For fish, we use the price of frozen fillets of sole, haddock, pollock, and halibut. The pricing study was conducted between 2014 and 2016, so we adjust for the change in the consumer price index of store food between 2016 and 2018 (0.989 (46); data for the NWT only available for Yellowknife).

### Carbon Emissions Data.

In assessing the emissions resulting from substituting traditional foods with market substitutes, it is our view that a comprehensive life-cycle approach that includes indirect emissions incurred in the production of food is most appropriate (*SI Appendix*). As above, we create three general categories of food products: beef and pork, poultry, and fish. For beef, pork, and poultry, we take the most recent “to-the-farmgate” emissions estimates from western Canada (47–49) (*SI Appendix*, Table S2). For the purpose of estimating carbon emissions of market substitutes for local fish, we use a mix of 70% common market whitefish (cod, pollock, haddock) and 30% salmon/trout, based on the rough proportions of the main fish species harvested in the ISR in 2018. We draw median emissions estimates for each of these groups of fish from a recent review (50). We convert the published estimates (per kilogram live or carcass weight) to bone-free meat weights and add average carbon costs per kilogram for processing, packing, and transport to retail distribution centers.

Finally, stores in the Inuvialuit region are exceptionally far away from major distribution centers. Food may travel to the Inuvialuit region through several different routes and modes of transport, including road, barge, and air freight. Carbon emissions of these different modes of transport depend on a wide range of conditions, including the size of the vehicles, engine type, river/sea conditions (in the case of barges), and so on. Given this variance in conditions, we adopt a simple approach in which we use high- and low-end estimates of carbon emissions (per kilogram per kilometer) for each mode of transport from the 2014 IPCC report on transport (51) (*SI Appendix*). At this stage, we consider direct carbon emissions only (i.e., fuel burned), not indirect emissions (e.g., produced in vehicle manufacturing). This is a conservative approach as it assumes that no additional vehicles would be built to transport additional imported food.

### Fuel Use Data.

Finally, we need a means to infer, from the available harvest data, cash and carbon inputs to harvest production. To do this, we use data on 132 harvesting trips recorded in the Tooniktoyok study, conducted with 10 harvesters in Ulukhaktok in 2019 (41, 42). This dataset contains estimates of fuel usage and harvested quantities. Usher’s edible weight estimates were used in the Tooniktoyok data, and so the edible weight data should be highly comparable to the IHS data. We note that in the Tooniktoyok interviews, interviewees generally reported their individual harvesting expenses, while harvests were often reported for groups of harvesters (e.g., if one caribou was caught). Consequently, to avoid overestimating the returns from harvesting when using these data, we take the estimates for individual harvests where provided, and where they were not, if it was a snowmobile or ATV trip, we divide the harvest by the number of participants on the trip. This is a conservative approach that is equivalent to assuming that each participant had their own machine. In contrast, for trips using boats, we include the total harvest, which assumes that only one boat (with multiple people in it) was involved in the reported catch.

### Analysis.

To calculate the edible harvest represented in the 2018 IHS, and to estimate its market value and carbon cost, we adopt a Bayesian approach that allows us to account for measurement error in the harvest reports and to account for unobserved processes in data generation (i.e., the absence of reports on failed harvesting trips in the IHS). The model has four main components: 1) we account for measurement error in the harvest data; 2) we run a regression model using the Tooniktoyok data that estimates i) fuel use per kilogram of edible weight, and ii) the proportion of trips that did not yield any harvest; 3) we use the posterior distribution of harvest sizes from step (1) to calculate the edible weight represented in the IHS and its associated market replacement costs and carbon emissions; and 4) we use the posterior distributions from steps (1) and (2) to calculate fuel usage represented in the IHS data and its associated gasoline consumption. We detail each of these steps below.

The first component of the model deals with measurement error in the IHS data. This involves accounting for the problem of “heaping” and, to a much lesser extent, imputation of missing harvest values. To account for the error in harvest estimates induced by heaping, we model each reported harvest as being taken from a lognormal distribution with a median equal to a latent variable representing the “true harvest” and a scaling factor of 0.15. The scaling factor was chosen on the basis of the relationship between the median and SD in lognormal distributions with different scaling factors. A scaling factor of 0.15 implies that a reported harvest of 10 items would have a SD of about 1.5 items, and a reported harvest of 500 items would have a SD of about 50 items (*SI Appendix*). This distribution accounts for the scaling of error with the size of the estimate (i.e., larger estimates are more heaped than smaller ones). We then model the true harvest as being taken from a probability distribution of harvest sizes which is unique for every “species type” within the dataset. We use the term species type because, for this procedure, we grouped multiple species into groups that are ecologically similar and harvested using similar techniques, and therefore are expected to have similar distributions of harvest sizes. For instance, we grouped several species of geese into a single group “geese,” and treated all subspecies of caribou as “caribou.” We model the log of the true harvest for each report in the IHS as drawn from a normal distribution with mean equal to the mean logged observed harvest size for that species type and with SD equal to the SD of the logged observed harvests for that species type. For missing data, we draw from this distribution to generate a predicted harvest value. Modeling true harvests from this species type distribution is a conservative approach that treats large harvest size estimates for a given species with greater skepticism. In *SI Appendix*, we consider an alternative approach that does not fit the true harvest estimates to a species-typical distribution, instead using them only to impute missing data.

The second step of the modeling procedure estimates gas consumption as a function of edible weight harvested and the frequency of unsuccessful trips, using the Tooniktoyok dataset. First, we model the probability that a trip was successful or not using a Bernoulli distribution with success probability θ, which is estimated from the data. For successful trips, we then model log fuel consumption as a linear function (intercept and slope) of log edible weight harvested. For unsuccessful trips, which returned no edible weight, we estimate the mean and variance in log fuel consumed using a normal distribution.

The third component of the model involves simulating true harvest amounts from the posterior distributions and multiplying each estimated harvest by the edible weight for the type of animal concerned (not pooled by species type). It is then straightforward to multiply these estimates by the per-kilogram cash and carbon costs of market replacements that we generated (above). We assign each reported harvest to the poultry, fish, or beef/pork category, and calculate sums of the estimates for each category. These operations are undertaken such that uncertainties at each step are fully propagated through to the final estimates. We do not account for any error or variation in edible weight; but our deheaping procedure results in fractional harvest counts, which accounts to some extent for variability in animal size and/or the portion used. Given the absence of available data, we do not account for measurement error in the market cost or carbon emissions estimates, although we do calculate estimates for different plausible scenarios for carbon emissions (*SI Appendix*).

Finally, the last component of the model uses the edible weight estimates from the previous step along with the parameters from the Tooniktoyok regression (step 2) to estimate the fuel used for each harvest reported in the IHS. A limitation here is that we could not reconstruct individual harvest trips from the IHS data, so we treat every harvest as a distinct trip. As such, our fuel use estimates may be somewhat high. We use θ, the probability of a failed trip, and the estimated mean and SD of log fuel use on failed trips from the Tooniktoyok model to infer the number of unobserved unsuccessful trips that are likely to be associated with the successful harvests observed in the IHS data and the fuel usage for these trips. To estimate the carbon emissions incurred from the shipping and burning of gasoline used in harvesting, we calculated high and low emissions scenarios for shipping gasoline to the ISR via rail to Hay River followed by barge to each community, converted kilograms to liters using a density of 0.749 kg/L, and then added emissions per liter shipping to the carbon emissions produced from burning a liter of gasoline (2.319 kg/L (52)).

The computer code for the analysis is included in *SI Appendix*. We used the R statistical computing environment (4.3.2) (53), Stan (2.32.2) (54), RStan (v2.32.5) (55), and the rethinking package (2.40) (56) for analysis. Our priors for all estimated parameters were mildly regulating, and are defined in the accompanying R code. The model was fit using Hamiltonian MCMC in the Stan engine, on three MCMC chains with 4,000 iterations each. Convergence was diagnosed by the Gelman–Rubin R-hat statistic, and manual inspection of traceplots to confirm that the chains were well mixed. Fig. 2 was produced using rphylopic (57).

### Inclusion and Ethics Statement.

The research reported here used only previously collected, deidentified data. A proposal for the article and the resulting manuscript were reviewed and approved by the IRC. The IRC, in collaboration with the Inuvialuit HTCs, designed and implemented the IHS. The Tooniktoyok Project was collaboratively designed and implemented by Angus Naylor and the project participants, and overseen by a volunteer Inuit Oversight Committee. The Tooniktoyok study protocols were approved by the research ethics boards of the University of Guelph (REB 17-12-012) and the University of Leeds (AREA 18-117), and the project was licensed by the Aurora Research Institute (No. 16533).

## Supplementary Material

Appendix 01 (PDF)

Dataset S01 (CSV)

## Data Availability

Access to the IHS data requires review by and permission from the Inuvialuit Regional Council and the Inuvialuit Game Council. Requests for access should be directed to the IRC Division of Innovation, Inuvialuit Science, and Climate Change (https://irc.inuvialuit.com/research/). Access to the Tooniktoyok Project dataset was provided by Angus Naylor and the Hamlet of Ulukhaktok with the permission of the Tooniktoyok Hunters. Data requests should be addressed to the Senior Administrative Officer, Hamlet of Ulukhaktok, sao_ulu@northwestel.net. The edible weight data, as well as our derivations of per kilogram market substitute costs and carbon emissions, are included in *SI Appendix*. R and Stan code for the analysis are available at https://github.com/elspethr/inuvialuit_carbon (58).
