# Supplementary material for: Indigenous food production in a carbon economy
Source: Proc Natl Acad Sci U S A. 2024 Jul 29;121(32):e2317686121. doi: 10.1073/pnas.2317686121 (PMC11317563; doi:10.1073/pnas.2317686121)
Supplement: Supplementary file 1 — Appendix 01 (PDF) [file pnas.2317686121.sapp.pdf]

Supplementary Information

Indigenous food production in a carbon economy

Elspeth Ready, Cody T. Ross, Bret Beheim and Jenn Parrott

S1. Additional discussion

Longitudinal trends. Past studies estimated the per capita harvest of country food in the ISR to be 116 kg/year from 1988 to 1997 (1). More recently, a 2018 study in Paulatuk estimated the total harvest in this community to be an average of 102.5 kg per capita (2). Our estimate for Paulatuk is 58.2 kg per Beneficiary, but the response rate in our data from Paulatuk was variable, with approximately 54–64% of Hunters and Trappers' Committee members reporting each month, and there was no harvest data from Paulatuk in February 2018. If we proportionally adjust our estimates to account for missingness, we obtain a per capita harvest of 96.0 kg in Paulatuk (see S2). Our estimate for Ulukhaktok, which had a relatively high coverage rate of harvesters, was 95.9 kg per capita. As such, although our estimates are lower than historical ones (due to our inability to extrapolate the IHS data), we do not think that they represent a substantial decline in total harvests; to the contrary, the data suggest that per capita harvests have likely remained relatively stable in recent decades.

An estimate of the replacement cost of traditional harvests in the ISR from 1988–1997 was $3.35 million annually, or $1150 per capita (1). Adjusted for inflation this would correspond to a total $4.91 million in 2018. The 1988–1997 study used a $10/kg food cost, which is considerably lower than the prices per kilogram used here. Our estimates represent a departure from past estimates, because we focus specifically on foods of similar kind (unprocessed meat) rather than the average cost of available market foods. We believe that our cost estimate is more appropriate, given that it compares matched food types and that there is well-established evidence that substitution of country food with cheaper, but nutrient-poor, foods is detrimental to Inuit health (3–9).

Limitations. Although we have used state-of-the-art statistical methods in generating our estimates, our results should nevertheless be treated with some caution. As mentioned in the main text and further explained below (see S2), our estimates of the number of animals harvested should be considered as minimum estimates. In our analysis, we also rely on the assumption that the Tooniktoyok data is representative of the relationships between fuel use and harvest production across communities in the ISR. While this dataset is currently the most fine-grained data available on inputs to and outputs of harvesting in the ISR, it is still based on a relatively small sample of harvesters and harvest trips and does not capture the entire range of harvest conditions encountered by Inuvialuit. Because the IHS data contains limited information on the context of harvesting, our regression approach was necessarily limited. Adding additional variables to the analysis (particularly what type of transportation was used) would likely considerably improve the accuracy of our fuel use estimates. Indeed, due to the difficulties involved in collecting data on inputs to harvesting, past assessments of subsistence production in the ISR have not attempted to measure them on regional scale (1). Despite these data limitations, modern Bayesian inference techniques have allowed us to take a first step in this direction.

In addition to the issues already mentioned, a further limitation of our study is that we have only considered one year of harvest data. Examining harvests over multiple years is important, as annual harvests may vary tremendously from year-to-year as a result of fluctuations in weather, animal movements, and other factors (10). Comments from participants in the IHS suggest that 2018 was a relatively poor harvest year in the region, especially for fish (11).

Our experience conducting this analysis leads us to offer two suggestions for researchers and agencies wishing to investigate the carbon inputs, outputs, or replacement value of Indigenous or other local, small-scale food systems. First, fine-grained, small-scale studies (e.g., the Tooniktoyok study) which

quantify inputs to local production (e.g., through variables like equipment longevity, rates of equipment ownership, and consumption of gasoline or other supplies) are extremely useful in the context of a Bayesian approach, as we are able to use fine-grained data to infer parameters that may be difficult to collect at the regional scale. Second, when collecting regional-scale data, sampling strategies that allow for reliable extrapolation to the whole population will generally offer more insight than sampling strategies which produce a larger volume of data without a well-defined sampling frame.

S2. The 2018 Inuvialuit Harvest Study

All Inuvialuit harvesters, who were 16 years or older and were registered with their local Hunters and Trappers' Committee (HTC), were asked to participate in the 2018 Inuvialuit Harvest Study (IHS). Harvest reports were collected by Community Resource Technicians (CRTs) who visited or contacted (e.g., by telephone) harvesters on a monthly basis. Generally, CRTs collected data in interviews at harvester's homes using paper record forms and an iPad. As an incentive to participate, each month participants were entered in a raffle to win a $50 gas or grocery card. The harvest data are collected anonymously; harvester names are never attached to harvester ID numbers.

During the monthly interviews, it was recorded whether study participants had harvested any animals in the past month, if they had gone out but not harvested anything, if they had not gone out, or if the harvester could not be interviewed or contacted that month. If the participant had harvested anything in past month, they were asked to report the type of animal, the number harvested, the sex of the animals (unknown, male or female), the maturity of the animals (unknown, young, juvenile or mature), their health condition (unknown, unhealthy, average, or very healthy), and the location and date for each successful harvest.

Despite the aim of regular sampling of all registered harvesters, response rates were highly variable both between and within communities (Figure S1). The upper panel in Figure S1 shows the proportion of HTC members in each community who were successfully contacted each month. The data do not reveal any consistent seasonal bias in response rates across the communities. However, there are three months for which there are no harvest data from Inuvik (January, February, and June). Overall, the response rates are almost always less than 60% of HTC members, and often even lower, which gives an idea of the potential scaling between the reported and total harvest.

The centre panel in Figure S1 shows the proportion of survey respondents each month who reported a catch. This is potentially revealing about sampling biases. If the survey was capturing a representative sample of harvesters each month, then we might expect a seasonal pattern to emerge, in which the proportion of harvesters reporting catches increases during the spring and summer and is lowest during the winter (because fewer harvesters are active in winter). This is to some extent visible in the data from Tuktoyaktuk, but in general is not evident, which suggests that active and inactive harvesters may have had different probabilities of being sampled across months.

We highlight trends in two villages that we think are particularly informative about patterns of harvesting in the region and the study sample. First, in Paulatuk, the proportion of harvesters sampled each month was highly consistent (besides the lack of data for February). Although it seems possible that highly active harvesters may have been somewhat undersampled relative to non-active harvesters in this community in some months (Figure S1 centre panel, June and August), it seems that extrapolating from the sample to the total harvest for this village is reasonable. If our estimates reflect approximately 60% of the total harvest in Paulatuk, the true per capita harvest estimate would be approximately 96.0 kg.

In comparison, in Ulukhaktok, response rates are inconsistent and decline towards the end of the year (Figure 2, top panel). In fact, after May, 100% of respondents in the Ulukhaktok sample each month reported catches (Figure 2, centre panel). What seems to have occurred is that as the year progressed, only active harvesters were recorded in the survey each month. The number of total harvesters reporting

catches in Ulukhaktok shows strong seasonal patterning (Figure 3, bottom panel) that is consistent with known seasonal trends in harvest activity. Overall, it seems likely that the low-season data for Ulukhaktok data are a nearly complete sample of active harvesters each month, while spring/summer harvests may be underestimated. We note that the per capita harvest recorded in Ulukhaktok is the highest of the villages at roughly 96 kg per beneficiary, an estimate similar to the value for Paulatuk we extrapolated earlier. In both of these cases these are still probably low estimates for the total harvests, as active harvesters were probably undersampled in some months.

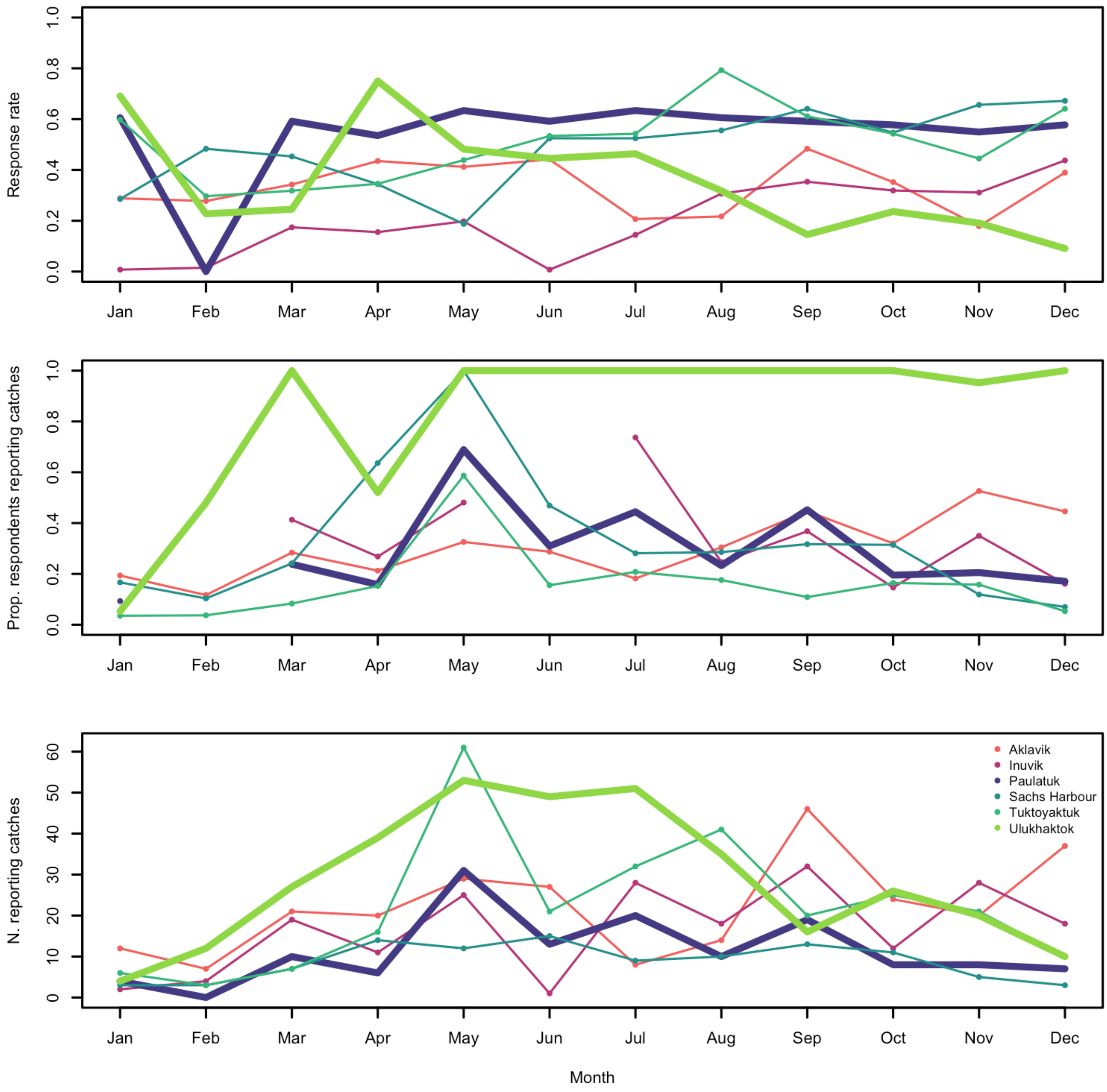

Figure S1. Monthly response rates in the 2018 Inuvialuit Harvest survey. Proportion of HTC members who responded to the survey each month (top panel), proportion (middle panel) and total number (bottom panel) of harvesters who reported catches each month. Lines for Paulatuk and Ulukhaktok are emphasized as the trends in these villages are particularly informative.

The above discussion highlights the difficulties in extrapolating from the reported to the total harvest: in some communities, the sample is biased towards active harvesters, but this bias varies from month to month due to seasonal changes in harvesting activities and other factors. Nevertheless, the average monthly participation rate of active harvesters was 36%, which suggests that the true harvest could be more than twice as much as reported (see 12 for a more in-depth discussion).

S3. Edible weight data

The edible weights we use to calculate food amounts from the number of animals harvested are provided as a supplementary data file. Here we provide a detailed explanation of our choices or derivations of edible weight values. For mammals, we adopt Usher's values (13), where available, as they are consistently lower and, we believe, more accurate than most estimates reviewed by Ashley (10). We avoided using averages of published estimates because many of these ultimately derive from only one or two original sources. We exclude species primarily harvested for pelts (mainly carnivores, including wolf, wolverine, fox, marten, mink, lynx, and otter, as well as squirrel). For beluga we include only the amount of muktuk acquired, as this is generally all that is used for human consumption in the Inuvialuit region.

Three mammals—bear, muskrat, and bearded seal—provide a particular challenge in terms of assessing edible weight because, although they are generally harvested for furs, they are also eaten with some regularity. Past analyses have not included these animals in estimates of the traditional food produced in the Inuvialuit Settlement Region (ISR) for this reason (1). In the case of bearded seals, which are taken very rarely in the ISR (only eight reported in the 2018 harvest data), they are generally harvested specifically for the purpose of obtaining materials for making traditional skin boots. Although part of the seal may occasionally be eaten, some bearded seals (particularly large males) are considered entirely inedible due to their strong taste and the meat is primarily used as dog food. Consequently, we omit bearded seals from our calculation of the edible harvest. Concerning bear, a 2008 report on local and traditional knowledge of grizzly bear indicated that Inuvialuit in Aklavik rarely eat grizzly bear (14), so we also do not include grizzly bear in the calculation of edible weights. However, a substantial portion of polar bear meat is consumed in some communities. For instance, in Ulukhaktok, parts of polar bear (especially the feet) are considered a delicacy, and when a bear is caught the meat is distributed to all households. Therefore, we use Usher's conservative estimate of 114 kg for polar bear (15). For muskrat, a 1992 study found that muskrat was among the preferred country foods among Inuvialuit in Aklavik and that households reported eating muskrat an average of 26 times per year (16). Even at a much lower frequency of consumption, this suggests that the entire muskrat harvest reported in Aklavik in 2018 (the focal point of muskrat harvesting in the ISR) could easily have been consumed.

For birds, we compared Usher's (13) edible weight values to the midpoint weight of all the bird species in the dataset from allaboutbirds.org, a bird identification resource maintained by the Cornell Lab of Ornithology. We derived new edible weight estimates by multiplying this midpoint weight by Usher's conversion factor of 0.7 (originally from 17). This method produced edible weight values extremely similar to Usher's, and so we used this method to impute edible weights for birds not included in Usher's dataset. However, we used Usher's values where available as these were often based on studies specific to the ISR. In one case (loon), we used a published value in the literature that was lower than our calculated value.

Finally, for fish, we use Usher's estimates where available, as these are based on local measurements, which are particularly important for fish given considerable within-species variation in size. For char, we use Usher's place-specific estimates for Ulukhaktok, Sachs Harbour, Paulatuk and Aklavik. We use the Aklavik value for Inuvik. We also use Usher's Aklavik char estimate (which presumably refers to the smaller Dolly Varden) for land-locked char. No char were harvested in Tuktoyaktuk. Where estimates from Usher were not available, we took edible weight estimates from a 2016 review of published estimates for a range of fish species harvested in Alaska (18). Finally, we derived new values for a small

number of species using empirically-derived length-weight equations for those species (available on fishbase.org). The species referred to as “Arctic cod” in the IHS is likely actually Greenland cod, called “ugoq” locally. We base our estimates for the weight of “Arctic cod” on a 40-cm Greenland cod.

The measures of edible weights in the literature are generally point estimates, and thus do not account for variation in animal size or in the portion of the animal that is consumed. Although it is standard practice in the literature on mixed economies in Northern Canada and Alaska, the use of single point estimates for edible weights of animals is potentially problematic. Harvested animals can be highly variable in size due to factors including age, sex, season, or the method or location of harvest. The portion of harvested animals that is actually consumed also varies widely, as a result of local tastes and habits, transport and storage constraints during harvesting trips, and the condition of the animal. A better approach to estimating edible weights would be to use a larger sample of measurements of harvested animals to estimate the distribution of harvested animal size and the variability in the portion used. Unfortunately, such information is extremely difficult to collect, and thus Usher’s data remain the best basis for the edible weights of many species in the dataset.

Finally no systematic harvest data on berries or other gathered resources (e.g., bird eggs) are available and as such these are also excluded from our estimations. We can, however, attempt a ballpark estimate of berry harvests using dietary recall studies from the 2007–2008 Inuit Health Survey, in which an average consumption of 2.2 ± 24 grams of local berries in 24-hr recall data were reported (19). If similar consumption rates occurred for the entire year (berries are frequently frozen and stored), annual berry consumption would be an average of 0.8 kg per person per year, suggesting an annual harvest of 2248 kg (for approximately 2800 Inuvialuit Beneficiaries).

S4. Substitution value of market products.

We focus on the substitution value of harvested foods, following Usher, who argues that “substitution costs provide the most appropriate measure of value [of country foods] and their use is, therefore, recommended” (20, pg. 105). Retail substitution costs are a welfare equivalent, that is, they measure the change in economic welfare of consumers as a result of a change in the market. Usher argues that welfare-equivalent measures are appropriate for the study of the impact of economic development on wildlife harvesting in northern Indigenous communities, as a result of a conflict between such development and the ability of Indigenous people to freely participate in traditional harvesting. Similarly, Brown and Burch argue that where society has a responsibility to provide a good (e.g., for equity reasons), “then the value to society of the wildlife unit is as great as the cost of replacing it, since society would incur that cost in the event of lack of availability of the good in question” (21, pg. 232). Although several decades old, these arguments hold in the context of carbon pricing: substantial increases in fuel potentially threaten the traditional harvesting activities of Inuvialuit, activities which are protected under the Inuvialuit Final Agreement. Besides a clear theoretical justification for using substitution value, data for other kinds of value (e.g., the market value) are simply not available.

Thus we focus on the retail substitution costs of market foods, albeit with the “caution that they cannot serve to measure the value of the activity or environment which produces the country food” (20, p. 105). The substitution value of harvested foods excludes any accounting for taste preferences, for instance. Most harvest production today is used for food (20), and we do not attempt to estimate the value of raw materials for clothing and craft production contributed through harvesting. Nevertheless, a large proportion of locally-produced clothing and arts and crafts are used by Inuvialuit (e.g., for coats and mittens) or sold on the informal market, and the economic importance of these activities is not well-represented in official statistics. We also do not attempt to estimate the more indirect or less tangible benefits of the traditional economy. We are not able, for instance, to determine the increased health care costs that would result from poorer nutrition or poorer mental health in Inuvialuit communities due to decreased availability of country food or participation in traditional activities.

To convert harvested kilograms into retail equivalents, we sum the estimated edible weights of all reported harvests in each community into three categories—mammals, poultry, and fish—and multiply these quantities by the price of similar replacement foods (i.e., unprocessed fresh or frozen meat products) in ISR communities. In consideration of the ways in which traditional foods are processed and consumed, and their high quality and nutritional value, we believe using the cost of high-quality unprocessed cuts of meat and fish provides the most appropriate comparison for country food. However, it should be emphasised that this does not represent what Inuvialuit would actually purchase if country food were not available, as these items would be prohibitively expensive for many Inuvialuit (19). Nor do these comparisons account for Inuvialuit tastes and preferences, because country foods are irreplaceable from this perspective.

Our price estimates of the cost per kilogram of replacement foods are shown in Table S1. These estimates are based on average prices reported in each ISR community from an in-store costing study that established lowest regular prices for preferred purchase volumes of a wide variety of foods (22). As a market substitute for locally-harvested mammals, we use a mix of 50% pork and 50% beef, based on historical patterns of beef and pork consumption in Canada (23), and consumption data that suggest that Inuvialuit consume relatively similar amounts of pork and beef (24). We take the average price of the three fresh/frozen cuts of beef (ground beef, steak, and roast) from (22) as the price equivalent for beef, and use pork chops for pork as this is the only type of unprocessed pork in the dataset. We used the price of an even mix of chicken legs and chicken breast as a comparison for birds, and the price of frozen fillets of sole, haddock, pollock, and halibut for fish. The latter was the only item in the dataset that we felt was a suitable comparison for local fish; we considered canned fish, which is considerably cheaper, to be an unsuitable comparison.

Table S1. Estimated 2018 price ($/kg) of retail substitutes

	Aklavik	Inuvik	Paulatuk	Sachs Harbour	Tuktoyaktuk	Ulukhaktok
**Beef and Pork (50/50 mix)**	**31.15**	**19.26**	**30.59**	**20.62**	**22.70**	**24.87**
Pork loin, center-cut chops, bone-in	28.64	14.38	27.87	18.33	19.64	24.92
Ground beef, lean	17.27	17.04	12.92	15.24	10.10	19.65
Beef round roast, inside (top)[1]	32.72	27.08	-	22.66	30.30	22.85
Beef steak, inside round	50.96	28.34	53.69	30.8	36.88	31.94
**Chicken**	**13.91**	**15.08**	**12.59**	**11.20**	**12.36**	**15.72**
Legs	11.71	11.36	8.77	6.73	9.24	13.08
Breast	16.11	18.80	16.04	15.68	15.49	18.35
**Fish (frozen fillets)**	**29.78**	**21.36**	**17.31**	**33.70**	**33.62**	**41.65**

1. No estimate for beef roast was available for Paulatuk so we used the average across all communities

Given the high cost of importing food, the more remote communities in the ISR receive additional subsidies from the federal government to offset these costs. Subsidies for transport of frozen and fresh meat by food mail (see the following section) to Aklavik, Paulatuk, Sachs Harbour, and Ulukhaktok in 2020 were $5.75, $4.95, $7.25, $5.65 per kilogram respectively (25). Replacing country foods in these communities with market foods would therefore not only entail the replacement cost that Inuvialuit would pay at the store—described above—but a considerable additional premium paid by the government. At the above subsidy level, the amount of food reported in the IHS for these villages would result in an additional cost of $394,546.

S5. Carbon costs of market substitution

An important consideration in calculating greenhouse gas emissions is whether only direct emissions need to be accounted for, or whether indirect emissions also should be included. Direct emissions result from

the industry itself, while indirect emissions incorporate emissions from inputs to the industry as well (26). For example, in the case of beef production, an approach incorporating indirect emissions would include not only the emissions from the cattle industry itself (e.g., methane emitted by cattle), but also the greenhouse gas emissions resulting from the agricultural industry dedicated to feed production. In assessing the emissions resulting from substituting traditional foods with market substitutes, it is our view that a comprehensive life-cycle approach that includes indirect emissions incurred in the production of food is most appropriate.

Point estimates of greenhouse gas emissions "to the farmgate" or to the point of carcass processing for livestock industries in Canada have been calculated for beef, pork, and poultry (27–34). The most recent year included in these studies was 2006 for poultry, and 2011 for beef and pork (Table S2). More recent data on agricultural greenhouse gas emissions are available from the Government of Canada (35), however, only estimates of direct emissions for livestock production are provided in these reports and as such these data are not suitable for our purposes.

The adjusted estimates in Table S2 estimate total carbon emissions of meat production through to the delivery of a packaged product to retail distribution centres. To calculate these values, we convert published estimates (per kilogram live or carcass weight) to bone-free meat weights and add average carbon costs per kilogram for processing, packing, and transport to retail distribution centres, based on published estimates (36). This approach assumes that the entire carbon emissions of pork and beef should be assigned to meat, and not to other products such as leather or manure, which is appropriate if one assumes that these products would not be used if meat were not being produced. This assumption is debated in the literature (e.g., 30), however, given the available data it allows us to generate the most comparable estimates across the animals considered. As a check on the quality of the point estimates we used (i.e., to make sure they were not outliers), we compared our calculated estimates to the range of carbon estimates for beef, pork, and poultry in a systematic review of life-cycle assessments for a broad range of fresh foods (36). The most recent Canadian estimates tend to fall in the mid-to-low end of carbon emissions estimates in the data reviewed in (36). As earlier, we assume a mix of 50% beef and 50% pork for mammals when calculating emissions based on the reported harvest.

In estimating the dollar value of fish produced in the ISR, we use the retail cost of frozen fish fillets because we felt this was the only reasonably-comparable product in the food cost dataset. However, in estimating the equivalent carbon savings we are able to be somewhat more specific. The majority of the Inuvialuit fish harvest is composed of broad whitefish, inconnu, char, and lake trout. Based on the rough proportions of these fish in the 2018 harvest, we take a mix of 70% common market whitefish (cod, pollock, haddock) and 30% salmon/trout for the purpose of estimating carbon emissions. We draw median estimates for each of these groups of fish from (36).

Table S2. CO_2-equivalent emission factors (kg CO_2-equivalents per kg boneless meat) for beef, pork, poultry and fish. LW = live weight, CW = carcass weight.

	Adj. Est.	Original Est.	Estimate description	Source
Beef	20.69	9.68 kg CO_2e/kg LW	Western Canada, 2011	Vergé et al. 2018 (31)
Pork	4.68	2.33 kg CO_2e/kg CW	Western Canada, 2011	Mackenzie et al. 2015 (33)
Chicken	2.69	1.06 kg CO_2e/kg LW	Western Canada, 2006	Vergé et al. 2009 (29)
Cod, pollock, haddock (median)	3.40	3.40 kg CO_2e/kg product	Barents Sea line-caught cod	Sund 2009 (37), reported in (36)
Salmon, trout (median)	3.47	2.10 kg CO_2e/kg LW	Canada, farmed salmon	Pelletier and Tyedmers 2007 (38), reported in (36)

Table S3. Approximate direct carbon emissions ranges for different modes of transport, in grams CO_2/tonne·km, from (39, Figure 8.6).

**Mode of transport**	**Low**	**High**
Diesel freight train	25	60
Large heavy-duty road vehicle	70	190
Barge	25	60
Short-haul belly-hold in passenger aircraft	800	2000

Food may travel to the Inuvialuit region through several different routes and modes of transport, including road, barge, and air freight. Carbon emissions of these different modes of transport depend on a wide range of conditions, including the size of the vehicles, engine type, river/sea conditions (in the case of barges), and so on. Given these variations, to approach this problem we adopt a simple approach based on high- and low-ranges of carbon emissions estimates for weight and distance shipped for each mode of transport (39) (Table S3). We chose belly-hold cargo for air freight, because air freight in the ISR is generally shipped in combined passenger/cargo planes. The estimates in Table S3 are for direct emissions only (e.g., fuel burned), not indirect emissions (e.g., due to vehicle manufacturing).

We consider two scenarios for food shipping to the region. The first scenario, "barge," is a lower-carbon scenario that involves food being shipped by truck from Edmonton to Hay River and then by barge to each of the ISR communities (except Aklavik, which is reached by ice road from Inuvik). We choose this scenario based on a 2010 report on transport in northern Canada, which indicated that although there is a rail line to Hay River, rail shipping is primarily used for fuel, while dry cargo is shipped to Hay River by truck (40). The second scenario, "food mail," involves food being shipped by truck from Edmonton to Inuvik and then by road (for Tuktoyaktuk) or by air to the other communities (40). This mode of transport increases the cost of transport considerably for communities with no or seasonally-limited road access (Aklavik, Paulatuk, Sachs Harbour, and Ulukhaktok). We also calculate transport emissions for shipping fuel by rail freight from Edmonton to Hay River, followed by barge to each of the communities (and a short distance by road for Aklavik). We use this scenario later to estimate carbon emissions of gasoline used in harvesting.

We approximated the distances travelled to each community using Google Maps (using straight-line distance from Inuvik for air travel) and other sources (40) (Table S4). We consider only one-way costs of transport to the communities. Table S5 shows high and low direct carbon emissions estimates for each scenario using the per kilometre emissions estimates from Table S3 and the distances in Table S4. The estimates range from a low of 0.11 kg CO_2/kg for the low range "barge" scenario estimate (to Inuvik) to as high as 1.95 kg CO_2/kg for the high range "food mail" estimate to Ulukhaktok. To estimate the carbon emitted through the production and transport of retail food substitutes to the ISR, we simply multiply the harvest estimates for each food type for each village by the carbon emission factors for each food type plus the community-specific transport emissions.

Our approach here has several limitations, many of which we have already highlighted. For instance, we have used general estimates of carbon emissions in transport rather than values specific to the vehicles, loads, and conditions involved in the transport of goods to the ISR. Our analysis also does not include the impacts of the wholesale or retail trade sectors. Finally, it should be noted that current emissions from shipping may also not be a good indicator of future emissions. For instance, low water levels on the Mackenzie River may compromise the river freight route and force an increased reliance on subsidized air freight or other, longer shipping routes for non-perishable foods that are currently delivered using barges. This means that the relative dollar and carbon savings from locally-harvested food may increase due to the impacts of climate change.

Table S4. Approximate distances of different shipping route segments for ISR communities. Edm = Edmonton; HR = Hay River.

	Rail Edm-HR	Road Edm-HR	Barge from HR	Road Edm-Inuvik	Air from Inuvik	Road from Inuvik
Aklavik	1300	1050	1600	3175	55	86
Inuvik	1300	1050	1600	3175		
Paulatuk	1300	1050	2300	3175	400	
Sachs Harbour	1300	1050	2200	3175	512	
Tuktoyaktuk	1300	1050	1800	3175		153
Ulukhaktok	1300	1050	2400	3175	670	

Table S5. Estimated direct carbon emissions (in kg) per kilogram shipped to each community in the ISR, for six scenarios (barge, food mail, rail, and high/low emissions for each).

Community	Barge (low)	Barge (high)	Food mail (low)	Food mail (high)	Rail (low)	Rail (high)
Aklavik	0.12	0.31	0.27	0.71	0.08	0.18
Inuvik	0.11	0.30	0.22	0.60	0.07	0.17
Paulatuk	0.13	0.34	0.54	1.40	0.09	0.22
Sachs Harbour	0.13	0.33	0.63	1.63	0.09	0.21
Tuktoyaktuk	0.12	0.31	0.23	0.63	0.08	0.19
Ulukhaktok	0.13	0.34	0.76	1.95	0.09	0.22

S6. Fuel use and emissions data

In approaching the question of harvest inputs for the ISR on a regional level, we are faced with considerable data gaps. Data on inputs to the traditional economy are more difficult to collect than harvest reports, partly because they may be difficult to remember and partly because many items circulate through non-monetary exchange. For instance, replacement parts from old snow machines may be exchanged freely among harvesters (41) and Inuit seamstresses may sew winter clothing without cost for family members who share food with them. Costs are thus distributed across social networks: those who paid for supplies or equipment may not be in the same household as the harvester who uses them.

Second, contemporary harvesting relies on a vast array of equipment, supplies, and local infrastructure; including snowmobiles and boats, fish hooks and bullets, knives, rope, ice-drills, camp stoves, jerry cans, CB radios, locally-made tents, cabins and workshops built by harvesters, and rubber boots and parkas, to list only a small portion of the kit required. Some of these items may be used for years, even decades, and thus it is not only difficult to obtain data from harvesters on expenditures on this wide array of items, but also necessary to get an idea of the lifespan of these objects.

Further, given the multiple kinds of value that land-based activities provide in Inuvialuit communities, the dollar and carbon cost of the equipment required for these activities should in principle not be allocated to harvest production alone. Many of these items should rather be considered essentials for full participation in local life and for wellbeing in ISR communities. Being on-the-land and harvesting, for instance, are among the few recreational activities available in small remote communities. Not having access to harvesting gear—which is already a challenge for many Inuit (42–44)—is a serious problem for Inuit welfare that will incur other costs (e.g., for healthcare).

Consequently, in our view, not all of the costs and emissions associated with harvesting gear and equipment should be allocated to food production. An appropriate approach might be to assign to food production only the increased costs/emissions resulting from food production compared to a hypothetical counterfactual where harvest equipment was only used for "recreational" or "cultural" purposes. In reality, however, land-based activities cannot be divided into "recreational," "cultural" or "productive" categories (45); indeed, part of the contribution of land-based activities to Inuit cultural identity and mental health is because the activities are economically productive (46). We do not know what level of harvest participation is needed to maintain the documented health and wellness benefits of land-based activities, other than to say that many Inuit already consider participation levels to be too low.

With all these empirical and theoretical difficulties in mind, it is clear why past studies have not attempted to quantify inputs to the subsistence economy on a regional scale. Nevertheless, we think it is important to provide some impression of the scope of expenses incurred by harvesters, as these costs are already burdensome for many Inuvialuit households and carbon pricing policy will increase these costs. With respect to carbon emissions, gasoline and vehicle production are the main sources of carbon-emissions in the traditional economy. Given the above considerations and data limitations, here we focus on gasoline used in harvesting trips. For a longer, more speculative, discussion of costs and carbon emissions incurred through the production and purchase of motor vehicles used in the IRC, see (12).

To estimate the gasoline used in harvest production, we use data on 132 harvesting trips from the Tooniktoyok (meaning "extreme determination") study conducted by Angus Naylor with 10 harvesters in Ulukhaktok in 2019 (see 47–48). Table S6 provides some summary statistics from this dataset, describing the gasoline inputs and other trip costs (e.g., ammunition, food, oil, naphtha; exclusive of major equipment costs). Table S7 summarises the posterior parameter estimates of our regression analysis of the Tooniktoyok data.

An important aspect of the Tooniktoyok dataset is that it includes information on unsuccessful harvesting trips. Roughly 20% of trips recorded were unsuccessful (21% in winter and 17% in summer), although it is possible that unsuccessful trips were less likely to be reported, so unsuccessful trips may actually be somewhat more frequent than suggested by the data. The information on unsuccessful trips is extremely important in estimating production costs, because the IHS data do not include information on how many unsuccessful harvest trips occurred. Any attempt to calculate harvest costs strictly from the reported IHS data would therefore miss the expenses incurred for unsuccessful trips.

Table S6. Summary of harvest trip expenditures by harvesters in the Tooniktoyok study (Ulukhaktok, 2019).

	Winter	Summer	Snowmobile	ATV	Boat	**All trips**
Gasoline volume (l)						
Min	2.27	1.45	2.27	1.45	7.96	**1.45**
Median	43.64	19.09	36.37	22.73	45.46	**36.37**
Mean	54.56	36.13	47.24	22.67	65.22	**47.12**
Max	227.30	250.03	227.3	68.19	190.94	**250.03**
Sample size	71	48	78	22	18	**119**
Total costs						
Min	6.31	3.37	6.31	3.37	41.05	**3.37**
Median	248.66	112.09	275.00	65.65	144.17	**168.44**
Mean	284.20	134.62	278.43	105.57	237.16	**238.87**
Max	1080.00	356.27	1080.00	356.27	776.68	**1080.00**
Sample size	46	20	41	12	13	**66**

Table S7. Posterior estimates of model parameters for regression analysis of fuel use in the Tooniktoyok dataset.

Parameter	Mean	St. Dev.
Theta (prob. failed trip)	0.244	0.040
Fuel intercept - successful	2.200	0.213
Fuel slope - successful	0.420	0.064
Standard deviation, linear estimator	0.831	0.065
Fuel intercept - failed trip	3.235	0.209
Fuel sd - failed trip	1.145	0.153

S7. Additional model outputs

Table S8. Posterior estimates of total edible weight (in kg), with village breakdown

	**Birds**			**Fish**			**Mammals**			**Total**		
	90% HPDI (low)	Mean	90% HPDI (high)	90% HPDI (low)	Mean	90% HPDI (high)	90% HPDI (low)	Mean	90% HPDI (high)	90% HPDI (low)	Mean	90% HPDI (high)
Aklavik	2287	2410	2531	4313	4657	4919	16510	17030	17524	23429	24097	24681
Inuvik	1337	1731	2119	4378	4646	4908	13174	13576	14023	19263	19953	20659
Paulatuk	3978	4120	4258	2756	2938	3113	6343	6534	6707	13296	13592	13880
Sachs Harbour	1076	1242	1385	601	758	911	1825	1970	2110	3672	3970	4245
Tuktoyaktuk	3511	3676	3823	16716	17355	18020	10822	11169	11498	31458	32199	32987
Ulukhaktok	2910	3032	3154	8714	8955	9171	15850	16318	16775	27762	28305	28829
Total	15641	16211	16747	38410	39308	40222	65668	66597	67477	120573	122117	123407

Table S9. Posterior estimates of cost of market substitutes (in Canadian dollars), with village breakdown

	**Birds**			**Fish**			**Mammals**			**Total**		
	90% HPDI (low)	Mean	90% HPDI (high)	90% HPDI (low)	Mean	90% HPDI (high)	90% HPDI (low)	Mean	90% HPDI (high)	90% HPDI (low)	Mean	90% HPDI (high)
Aklavik	31810	33518	35201	128455	138690	146479	514275	530498	545859	683419	702705	721352
Inuvik	20155	26109	31951	93516	99232	104835	253722	261474	270086	373967	386815	398993
Paulatuk	50087	51874	53609	47711	50855	53888	194037	199867	205181	295873	302597	309187
Sachs Harbour	12053	13916	15515	20244	25531	30688	37638	40626	43513	73326	80073	86801
Tuktoyaktuk	43390	45431	47254	561998	583461	605844	245663	253536	261009	859138	882427	905861
Ulukhaktok	45752	47660	49589	362935	372986	381975	394180	405826	417197	810356	826471	841062
Total	210343	218506	226056	1241336	1270756	1299060	1668580	1691827	1715630	3141689	3181089	3218131

Table S10. Posterior estimates of carbon emissions (tonnes CO_2-equivalents) of market substitutes, with village breakdown

	Birds			Fish			Mammals			Total		
	90% HPDI (low)	Mean	90% HPDI (high)	90% HPDI (low)	Mean	90% HPDI (high)	90% HPDI (low)	Mean	90% HPDI (high)	90% HPDI (low)	Mean	90% HPDI (high)
**Barge shipping (low emissions projections)**												
Aklavik	6	7	7	15	16	17	211	218	224	235	241	248
Inuvik	4	5	6	15	16	17	169	174	179	189	195	201
Paulatuk	11	12	12	10	10	11	81	84	86	103	106	108
Sachs Harbour	3	4	4	2	3	3	23	25	27	29	31	33
Tuktoyaktuk	10	10	11	59	61	64	139	143	147	210	215	220
Ulukhaktok	8	9	9	31	32	33	203	209	215	243	250	255
Total	44	46	47	136	139	143	841	853	864	1026	1038	1050
**Barge shipping (high emissions projections)**												
Aklavik	7	7	8	16	17	18	215	221	228	239	246	253
Inuvik	4	5	6	16	17	18	171	176	182	193	199	204
Paulatuk	12	12	13	10	11	12	83	85	87	106	109	111
Sachs Harbour	3	4	4	2	3	3	24	26	27	30	32	34
Tuktoyaktuk	11	11	11	62	65	67	141	145	149	216	221	226
Ulukhaktok	9	9	10	33	34	35	206	213	219	249	256	261
Total	47	49	50	144	147	150	854	866	877	1050	1062	1074
**Food mail shipping (low emissions projections)**												
Aklavik	7	7	7	16	17	18	214	221	227	238	245	251
Inuvik	4	5	6	16	17	18	170	175	181	191	197	203
Paulatuk	13	13	14	11	12	12	84	86	89	109	111	114
Sachs Harbour	4	4	5	2	3	4	24	26	28	31	33	36
Tuktoyaktuk	10	11	11	61	63	66	140	144	149	213	218	223
Ulukhaktok	10	10	11	36	37	38	213	219	226	261	267	274
Total	49	51	53	146	150	153	860	872	883	1060	1073	1085
**Food mail shipping (high emissions projections)**												
Aklavik	8	8	9	18	19	20	221	228	235	249	256	263
Inuvik	4	6	7	18	19	20	175	180	186	198	205	211
Paulatuk	16	17	17	13	14	15	89	92	94	120	123	126
Sachs Harbour	5	5	6	3	4	5	26	28	30	35	37	40
Tuktoyaktuk	12	12	13	68	70	73	144	149	153	226	231	236
Ulukhaktok	14	14	15	47	48	49	232	239	246	294	301	308
Total	60	62	64	171	174	178	904	916	929	1140	1153	1166

S8. Alternative model results

In the model presented in the main text of the paper, the log reported harvest for each report is modelled from a normal distribution with mean equal to the log "true harvest" (a latent variable representing the "true" number of animals harvested on a trip) and scaling factor 0.1. This step is intended to model the error produced by heaping in reporting. We further model the log of each "true harvest" event as coming from a normal distribution with the mean and standard deviation of the logged data for all the reported harvests of the "species-type" involved (see Methods). This modelling step means that very large harvests for a given type of animal are treated with more skepticism, such that for larger harvests, the model's estimate of the "true harvest" will likely be less than the reported value. Although we do not have empirical data to test our assumption, we believe that downscaling the estimated size of large catches is reasonable, due to potentially larger error in reporting and because the proportion of large catches consumed may be less than for small catches, due to factors such as transport capacity or labour constraints.

To examine the impact of this modelling step on the results, we also ran the model without the "species-type" distributions, instead only using these for the imputation of missing data (64 of 2,388 harvest reports). This alternative approach led to an estimated harvest of 124,679 ± 959 kg (mean, sd) and gasoline usage of 167,955 ± 22,417 litres, compared to the original model estimates of 122,116 ± 886 kg for the harvest and 167,361 ± 21,957 litres of gasoline. The difference in the results of the two modeling approaches is therefore relatively small, and the results presented in the main text reflect a more conservative estimate of the reported harvest, its replacement costs, and associated fuel usage.

S9. Robustness checks

To examine the robustness of our model, we wrote R code to generate simulated harvest trips for a subset of species in the dataset, starting by generating fuel quantities from a beta distribution. The simulation code then generates "true" harvest amounts as a function of fuel usage, and heaps these values to produce an "observed" harvest amount. We then ran our model on harvest data simulated using a wide range of parameter combinations, to determine whether the model could consistently recover the total fuel use and total harvest amount represented in the simulated data.

Overall, our model performed very well on the simulated data, generating total harvest estimates that were very close to the true total harvest value, with a slight tendency to underestimate the harvest. True fuel usage was contained within the 89% highest posterior density interval (HPDI) of the model output in most simulation runs. The code to produce simulated harvest data and to run the model on the simulated data are available at https://github.com/elspethr/inuvialuit_carbon.

References

1. P. J. Usher, Inuvialuit use of the Beaufort Sea and its resources, 1960-2000. *Arctic* **55(S1)**, 18–28 (2002).
2. C. Mussells, " 'Better than beef or chicken.' Household characteristics and country food system resilience in Paulatuk, NT." Master's thesis, University of Ottawa, Ottawa (2018).
3. H. V. Kuhnlein, O. Receveur, R. Soueida, R., G. M. Egeland, Arctic Indigenous peoples experience the nutrition transition with changing dietary patterns and obesity. *J. Nutr.* **134(6)**, 1447–1453 (2004).
4. M.-L. Chateau-Degat *et al.,* Cardiovascular burden and related risk factors among Nunavik (Quebec) Inuit: Insights from baseline findings in the Circumpolar Inuit Health in Transition cohort study. *Can. J. Cardiol.* **26(6)**, e190–e196 (2010).

5. B. Hopping, *et al.*, Socioeconomic indicators and frequency of traditional food, junk food, and fruit and vegetable consumption amongst Inuit adults in the Canadian Arctic. *J. Hum. Nutr. Diet.* **23**, 51–58 (2010).
6. G. M. Egeland, *et al.*, Food insecurity and nutrition transition combine to affect nutrient intakes in Canadian Arctic communities. *J. Nutr.* **141(9)**, 1746–1753 (2011).
7. N. Zienczuk, T. K. Young, Z. R. Cao, G. M. Egeland, Dietary correlates of an at-risk BMI among Inuit adults in the Canadian High Arctic: Cross-sectional International Polar Year Inuit Health Survey, 2007-2008. *Nutr. J.* **11(1)**, 1–8 (2012).
8. S. Bruce, N. Riediger, L. Lix, Chronic disease and chronic disease risk factors among First Nations, Inuit and Métis populations of northern Canada. *Chronic Dis. Inj. Can.* **34(4)**, 210–217 (2014).
9. T. Sheehy, F. Kolahdooz, C. Roache, S. Sharma, Traditional food consumption is associated with better diet quality and adequacy among Inuit adults in Nunavut, Canada. *Int. J. Food Sci. Nutr.* **66(4)**, 445–451 (2015.
10. B. Ashley, "Edible weights of wildlife species used for country food in the Northwest Territories and Nunavut." (Wildlife and Fisheries Division. Department of Resources, Wildlife and Economic Development, Government of the Northwest Territories, 2002).
11. IHS [Inuvialuit Harvest Study]. "Inuvialuit Harvest Study 2018 Partner Report." (Inuvialuit Settlement Region Community-Based Monitoring Program, The Inuvialuit Regional Corporation, Joint Secretariat and The Inuvialuit Game Council, 2019).
12. E. Ready, "Impacts of carbon pricing on the hunting, fishing and trapping economy in the Inuvialuit Settlement Region." (Inuvialuit Regional Corporation, Innovation, Inuvialuit Science & Climate Change Division, 2021). Available at: https://irc.inuvialuit.com/wp-content/uploads/2023/10/HFT_carbon_report.pdf (accessed 6 July 2024).
13. P. J. Usher, "Standard edible weights of harvested species in the Inuvialuit Settlement Region." (Northern Contaminants Program, Department of Indian Affairs and Northern Development, 2000).
14. Wildlife Management Advisory Council (North Slope) & the Aklavik Hunters and Trappers Committee, "Aklavik local and traditional knowledge about grizzly bears of the Yukon North Slope: Final report." (Wildlife Management Advisory Council (North Slope), 2008).
15. P. J. Usher, "The Bankslanders: Economy and ecology of a frontier trapping community." PhD dissertation, University of British Columbia, Vancouver (1971).
16. E. E. Wein, M. M. Freeman. Inuvialuit food use and food preferences in Aklavik, Northwest Territories, Canada. *Arctic Med. Res.* **51(4)**, 159–172 (1992).
17. T. E. White, A method of calculating the dietary percentage of various food animals utilized by Aboriginal peoples. *Am. Antiq.* **18(4)**, 396–398 (1953).
18. C. L. Brown, *et al.*, "Harvests and uses of wild resources in 4 Interior Alaska communities and 3 Arctic Alaska communities, 2014." (Technical Paper No. 426, Division of Subsistence, Alaska Department of Fish and Game, 2016).
19. T.-A. Kenny, *et al.*, Dietary sources of energy and nutrients in the contemporary diet of Inuit adults: Results from the 2007–08 Inuit Health Survey. *Pub. Health Nutr.* **21(7)**, 1319–1331 (2018).
20. P. J. Usher, Evaluating country food in the northern native economy. *Arctic* **29**, 105–120 (1976).
21. T. C. Brown, E. S. Burch Jr., "Estimating the economic value of subsistence harvest of wildlife in Alaska." in *Valuing wildlife resources in Alaska*, G. Peterson, C. S. Swanson, D. W. McCollum, M. H. Thomas, Eds. (Westview Press, Prospect Heights, 1992), pp. 203–254.
22. T.-A. Kenny, *et al.*, Calories are cheap, nutrients are expensive—the challenge of healthy living in Arctic communities. *Food Policy* 80, 39–54 (2018).
23. Government of Alberta, "Per capita consumption of meats in Canada and United States." Available at: https://open.alberta.ca/dataset/per-capita-consumption-of-meats-in-canada-and-united-states-1985-2014/resource/f9d4adbb-9004-48c3-b190-27945dc6d757. Last updated February 2, 2017.
24. T.-A. Kenny, "The Inuit food system: Ecological, economic and environmental dimensions of the nutrition transition." PhD dissertation, University of Ottawa, Ottawa (2017).

25. Government of Canada, "Eligible communities. Nutrition North Canada." Available at: https://www.nutritionnorthcanada.gc.ca/eng/1415540731169/1415540791407#wb-auto-4 (accessed 30 March 2021).
26. D. Pandey, M. Agrawal, J. S. Pandey, Carbon footprint: Current methods of estimation. *Environ. Monit. Assess.* **178(1)**, 135–160, (2011).
27. X. Vergé, J. Dyer, R. Desjardins, D. Worth, Greenhouse gas emissions from the Canadian beef industry. *Agri. Syst.* **98(2)**, 126–134 (2008).
28. X. Vergé, J. Dyer, R. Desjardins, D. Worth, Greenhouse gas emissions from the Canadian pork industry. *Livestock Sci.* **121(1)**, 92–101 (2009).
29. X. Vergé, J. Dyer, R. Desjardins, D. Worth, Long-term trends in greenhouse gas emissions from the Canadian poultry industry. *J. Appl. Poultry Res.* **18(2)**, 210–222 (2009).
30. X. Vergé, D. Maxime, R. Desjardins, A. VanderZaag. Allocation factors and issues in agricultural carbon footprint: A case study of the Canadian pork industry. *J. Clean. Prod.* **113**, 587–595 (2016).
31. X. Vergé, A. VanderZaag, R. Desjardins, B. McConkey, Synergistic effects of complementary production systems help reduce livestock environmental burdens. *J. Clean Prod.* **200**, 858–865 (2018).
32. R. L. Desjardins, *et al.*, Carbon footprint of beef cattle. *Sustainability* **4(12)**, 3279–3301 (2012).
33. S. Mackenzie, I. Leinonen, N. Ferguson, I. Kyriazakis, Accounting for uncertainty in the quantification of the environmental impacts of Canadian pig farming systems. *J. Anim. Sci.* **93(6)**, 3130–3143 (2015).
34. G. Legesse, *et al.*, Greenhouse gas emissions of Canadian beef production in 1981 as compared with 2011. *Anim. Prod. Sci.* **56(3)**, 153–168 (2016).
35. Environment and Climate Change Canada, "National Inventory Report 1990–2018: Greenhouse Gas Sources and Sinks in Canada." (Environment Canada, 2020). Available at: http://www.publications.gc.ca/site/eng/9.506002/publication.html.
36. S. Clune, E. Crossin, K. Verghese, Systematic review of greenhouse gas emissions for different fresh food categories. *J. Clean. Prod.* **140**, 766–783 (2017).
37. V. Sund, "Environmental assessment of Northeast Arctic cod caught by long-lines and Alaska pollock caught by pelagic trawls." (SIK Report No 799, The Swedish Institute and Food and Biotechnology and University of Gothenburg, 2009).
38. N. Pelletier, P. Tyedmers, Feeding farmed salmon: Is organic better? *Aquaculture* **272**, 399–416 (2007).
39. R. Sims, *et al.*, "Transport." in *Climate Change 2014: Mitigation of climate change. Contribution of Working Group III to the fifth assessment report of the Intergovernmental Panel on Climate Change*, O. Edenhofer, *et al.*, Eds. (Cambridge University Press, Cambridge, 2014).
40. PROLOG Canada Inc., in association with EBA Engineering Consultants Ltd., "The Northern Transportation Systems Assessment. Phase 1 Report: Northern Transportation Demand Assessment." (Transport Canada, 2010). Available at: https://buyandsell.gc.ca/cds/public/2018/04/11/786956da5aabf5eb7d35d624b80ad57a/ntsa_phase_1-_final-_word_version.pdf (accessed 31 March 2021).
41. P. Collings, Economic strategies, community, and food networks in Ulukhaktok, Northwest Territories. *Arctic* **64**, 207–219 (2011).
42. D. Natcher, S. Shirley, T. Rodon, C. Southcott, Constraints to wildlife harvesting among Aboriginal communities in Alaska and Canada. *Food Sec.* **8(6)**, 1153–1167, (2016).
43. E. Ready, Challenges in the assessment of Inuit food security. *Arctic* **69**, 266–280 (2016).
44. E. Ready, P. Collings, "All the problems in the community are multifaceted and related to each other": Inuit concerns in an era of climate change. *Am. J. Hum. Biol.* **33(4)**, e23516 (2021).
45. R. G. Condon, P. Collings, G. Wenzel, The best part of life: Subsistence hunting, ethnicity, and economic adaptation among young adult Inuit males. *Arctic* **48**, 31–46 (1995).
46. P. Collings, *Becoming Inummarik: Men's lives in an Inuit community.* (McGill-Queen's University Press, Montreal, 2014).

47. A. W. Naylor, *et al.*, Monitoring the dynamic vulnerability of an Arctic subsistence food system to climate change: The case of Ulukhaktok, NT. *PloS ONE* **16(9)**, e0258048 (2021).
48. A. W. Naylor, *et al.*, Understanding determinants of hunting trip productivity in an Arctic community. *Front. Sustain. Food Syst.* **5**, 688350 (2021).
